# Optimized Ultrasound-Assisted Extraction of Lignans from *Linum* Species with Green Solvents

**DOI:** 10.3390/molecules27092732

**Published:** 2022-04-23

**Authors:** Michela Alfieri, Iride Mascheretti, Roméo A. Dougué Kentsop, Monica Mattana, Marina Laura, Gianluca Ottolina

**Affiliations:** 1 Institute of Chemical Sciences and Technologies “Giulio Natta”, National Research Council, Via Mario Bianco 9, 20131 Milan, Italy; michela.alfieri@scitec.cnr.it; 2 Institute of Agricultural Biology and Biotechnology, National Research Council, Via Bassini 15, 20133 Milan, Italy; iride.mascheretti@ibba.cnr.it (I.M.); romeo.dougue@ibba.cnr.it (R.A.D.K.); monica.mattana@ibba.cnr.it (M.M.); 3 CREA Research Centre for Vegetable and Ornamental Crops (CREA OF), Corso degli Inglesi 508, 18038 Sanremo, Italy; marina.laura@crea.gov.it

**Keywords:** lignans, *Linum*, green extraction, green solvent

## Abstract

Lignans are plant phenols derived from phenylpropanoids. They play a significant role in plant defense and have features that make them appealing for pharmaceutical applications. Lignans can be obtained by plant in vitro cultures; their production by adventitious and hairy roots of *Linum* species seems to be a promising alternative to chemical synthesis. In the context of large-scale production, it is necessary to optimize their extraction from plants tissue by choosing the more suitable solvent and extraction procedure, paying attention to the use of green media and methods. With the aim to select the best conditions for the extraction of two interesting lignans (justicidin B and 6-methoxypodophyllotoxin) from *Linum* tissues, different green solvents and the method of ultrasound-assisted extraction were tested. The results showed that ethyl methyl ketone and dimethyl carbonate were the best media to extract justicidin B and 6-methoxypodophyllotoxin, respectively, in terms of purity and recovery. Moreover, we showed that ultrasound-assisted extraction presents different advantages compared to conventional methods. Finally, the optimal experimental conditions to extract justicidin B from *L. austriacum* hairy roots using methyl ethyl ketone were also determined by the response surface method. The models obtained are reliable and accurate to estimate the purity and recovery of justicidin B.

## 1. Introduction

Lignans are a class of secondary metabolites produced by several plant species, with a plant defense function. They are present in the flowering aerial part, seeds and roots [1,2,3]. Due to their biological activities (antiviral, cytotoxic, antioxidant), they are also valuable for human health [4]. Despite recent advances in lignans chemical synthesis [5,6], their high production time and costs have prompted the search for alternative options. Moreover, as the large-scale production of these compounds from plants is limited, an interesting alternative could be their production from cell cultures, adventitious roots or hairy roots, characterized by high genetic stability, biosynthetic capabilities, biomass production and the possibility to be used for several successive generations [7,8]. Based on their chemical structure, lignans are divided into several main classes [3]. In particular, aryltetralin-type lignans have attracted attention since the most representative compound podophyllotoxin is the key precursors of effective anticancer drugs such as etoposide, teniposide and other derivatives [9]. Other aryltetralin compounds interesting for their cytotoxic activity are 6-methoxypodophyllotoxin (MPTOX) and 6-methoxypodophyllotoxin-7-O-β-glucoside (MPTOX―Glc) [10]. These compounds are also present in the *Linum* species. The *Syllinum* section of the genus *Linum* comprises several species, among which *L. flavum, L. album* and *L. mucronatum* are the most investigated [11,12,13]. In a recent study, the adventitious roots of *L. dolomiticum*, in the *Syllinum* section, were reported to be good producers of MPTOX and MPTOX-Glc [14].

The section *Linum* accumulates predominantly arylnaphthalene-type lignans, of which, one of the most important is justicidin B (JB), which has antifungal, antiviral, antiparasitic, piscicidal, antiplatelet, anti-inflammatory and cytotoxic properties [15]. *L. austriacum* is one of the most intensively studied JB-producing species, whose cell and root cultures have been extensively described [16].

In a context of increased interest in these molecules and in view of their large-scale production in bioreactors, it is necessary to maximize their recovery from plant tissues. In general, the extraction yield of secondary metabolites from plants is dependent on the solvent and on the extraction method. In particular, the nature of the solvent is considered the most critical. Organic solvents such as methanol, ethanol, acetone and ethylene glycol and their aqueous solutions are commonly used [17,18]. However, due to the varied physical and chemical natures of the components present in plant materials, it is often unclear which solvent is the most effective. A sustainable process is desirable, and green solvents have been used as promising environmentally friendly media capable of replacing conventional ones. In the past few years, a new class of green solvents, deep eutectic solvent (DES), have also been considered for the extraction of bioactive compounds from a vegetal matrix due to their low toxicity and biodegradable nature [19,20].

Besides the properties of the solvents, extraction efficiency is also affected by the extraction method. Various extraction methods have been proposed, such as microwave-assisted extraction, ultrasonic-assisted extraction and supercritical fluid extraction [21]. Ultrasonic-assisted extraction (UAE) has already been used in the food industry and to extract phytochemical ingredients because, compared to other methods, it is eco-friendly and cost-effective and requires relatively less time, energy and solvents. It also poses a low physical risk and enhances the extraction quality [22].

The aim of this study was to identify the best conditions for the extraction of two representative lignans from plant tissue matrices, considering both recovery and purity of these molecules. Hence, several solvents and extraction methods—UAE, conventional extraction (CE: maceration)—were tested. Finally, after selecting the best solvent and extraction methods, a response surface methodology was used to describe and then improve the operational conditions, so to maximize JB recovery and purity.

## 2. Results and Discussion

### 2.1. Green Solvents and Extraction

In typical extraction and separation procedures, large quantities of organic solvents are used, but due to their toxic effects on human health and the environment, it is necessary to replace them with greener alternatives [23]. In this context, a different class of green solvents could be used, such as renewable, natural and deep eutectic solvents [24]. Beside the choice of solvents, the improvement of natural products extraction also involves the choice of the method of extraction. Ultrasonication generates the phenomenon of cavitation when the acoustic power input is sufficiently high to allow the production of multiple microbubbles and their violent collapse. The treatment of pulverized plant material with ultrasounds gives rise to a rapid disruption of the cell walls and membranes [22]. For this reason, nowadays UAE is a well-established technique commonly used to facilitate the extraction of any type of plant matrices and has been used in the food industry and to extract phytochemicals such as phenols [25,26] and lignans [21,27]. As an example of process intensification, the industrial extraction of the secondary metabolite artemisinin from *Artemisia annua* has been improved through the optimization of different extraction and purification methods using different green solvents [28]. Although, at an industrial level, it is preferred to use fresh or dried tissue, in this study the extraction was carried out on freeze-dried powdered tissue, since it is a more homogeneous material in terms of lignans content.

A panel of green solvents, except chloroform that was used here for comparison with other studies, with different chemical physical properties was tested (Table 1). These solvents are considered green in several solvent selection guides [24,29,30] and can cost up to four times more than methanol. In addition to conventional green solvents, two hydrophobic natural deep eutectic solvent (NADES) with food-grade components were also evaluated [19]. The use of this NADES was previously reported for the extraction of pesticides and contaminants from water and soil [31,32]. Regarding the miscibility in water, only 1,3-dioxolane, 3-methoxy-3-methylbutan-1-ol and methanol are reported to be completely miscible.

### 2.2. Justicidin B Extraction from L. austriacum Hairy Root Cultures

Nowadays, among plant tissue cultures as biological matrices for producing valuable metabolites, hairy root cultures (HRc), obtained by transforming plant tissues with *Agrobacterium rhizogenes*, are considered an interesting material, as they have high genetic stability and relatively fast growth rates (compared to normal roots) [33]. Hairy roots of *L. austriacum*, rich in JB (Figure S1) [16], were used to verify which solvent and extraction method was most effective.

**Figure 1:**
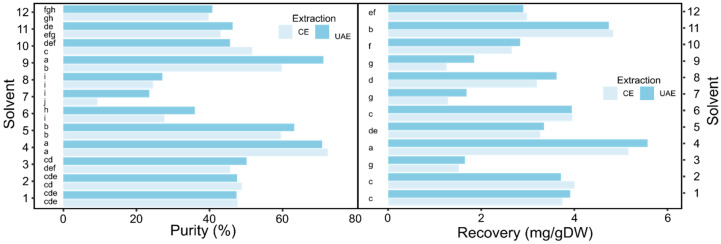
Mean of JB purity (**right**) and recovery (**left**) from *L.* *austriacum* HRc using UAE and CE. The samples with different letters are significantly different at *p* ≤ 0.05 according to Duncan test. Coefficient of variation (CV) purity = 4.9%; recovery = 7.3%. n = 3.

In general, adventitious and hairy roots cultures of *Linum* species produced variable amounts of JB in relation to the species, the type of tissue and the growth conditions, usually in the range of 5–20 mg/g dry weight (DW) of biomass [15]. In previous works, JB and other interesting lignans were extracted from plant tissues by means of a mixture of methanol and water (80/20, *v*/*v*) that, together with ethanol, are the benchmark solvent for polyphenols extraction [16,34,35]. In order to verify if the solvents and the extraction methods used affected the results, the data obtained in this study (Figure 1) were subjected to analysis of variance (ANOVA, as shown in the Table S1) and multiple comparison tests (Duncan, Figure 1 and Tuckey HSD, Figures S3 and S4).

For JB purity, both factors (solvents and extraction methods) and their interaction were found to be significant (as shown in Table S1). The choice of the medium affected purity more than the extraction method. Indeed, the F values in two-way ANOVA were 194.6 and 24.9, respectively (see Table S1). We found a small difference between the average values of JB purity obtained using UAE and those obtained using CE. For six solvents (3, 6, 7, 9, 11, 12), UAE allowed obtaining a high degree of JB purity as compared to CE (Figure 1). Solvents 4, 5 and 9 appeared to be the most suitable to extract JB in high purity. Conversely, for JB recovery, only the nature of the medium was found to be significant, while the extraction method (UAE vs. CE) did not affect JB recovery (Table S1). Therefore, UAE had the advantage of saving time (few minutes vs. overnight) with respect to CE. In contrast, solvents 4 and 11 were the most suitable to extract JB in high amount. In general, the use of a pure solvent without the addition of water is not recommended, since water promote the swelling of the dry biomass and, therefore, the access of the solvent, favoring the solubilization of lignans. However, the use of two pure green solvents (3 and 7) was tested and JB recovery was, as expected, below average (about 1.5 mg/g DW). A similar recovery value was obtained using chloroform (solvent 9).

**Figure 2:**
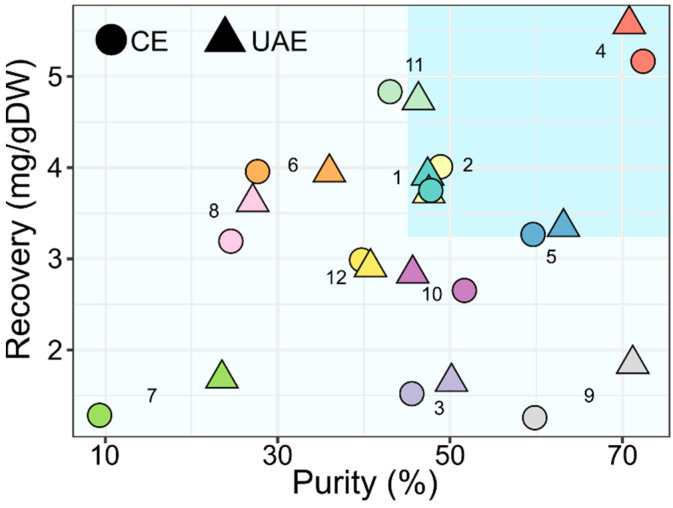
Purity–recovery graph for JB extraction. Purity is expressed as percentage (%), recovery is expressed as milligrams for gram of dry weight (mg/g DW). In the top-right section, extractions above the average within the solvents employed are shown.

The choice of the best solvent and extraction method must take in account both parameters, i.e., recovery and purity. In fact, a high recovery allows increasing the yield and decreasing the waste, and a high purity facilitates the purification process, reducing the downstream costs. The purity–recovery graph of JB is shown in Figure 2.

The recovery and purity average values were 3.2 mg/g DW and 45.8 %, respectively. The solvents with recovery and purity values above the average were 1, 2, 4 and 5 with both extraction methods and 11 only when using UAE (Figure 2). Solvent 11, the deep eutectic solvent derived from the combination of menthol and decanoic acid (Table 1), showed a good performance in terms of extraction. In general, the application of this kind of solvents (like ionic liquid solvents) for bioactive compounds extraction form a natural matrix presents the problem of recovering the extracted metabolite, since the simple evaporation step cannot be applied. Indeed, different solutions have been proposed, such as precipitation and separation with a chromatographic column or by means of liquid–liquid extraction, but they cannot always be applied, also due to their increased procedural times and costs [36,37]. In the end, the best solvent that allowed maximizing JB purity and recovery was solvent 4, regardless of the extraction method, because no significant difference was found between the two types of extraction (see Duncan test in Figure 1 and Tukey test in Figures S3 and S4).

However, it has been reported that ultrasound leads to a greater decrease in particle size than conventional extraction methods and enhances the mass transport kinetics, contributing to the decrease in the time required for the extraction [22]. In our experiments, extraction required few minutes, whereas overnight incubation was necessary in the case of CE. Regarding the extraction with 80% methanol, solvent 4 allowed recovering about 30% more JB which was 33% purer; these values can offset the higher cost of ethyl methyl ketone. The high value of the compound combined with its high degree of purity and yield can justify the solvent and energy costs for its extraction.

### 2.3. MPTOX Extraction from L. dolomiticum Adventitious Root Cultures

**Figure 3:**
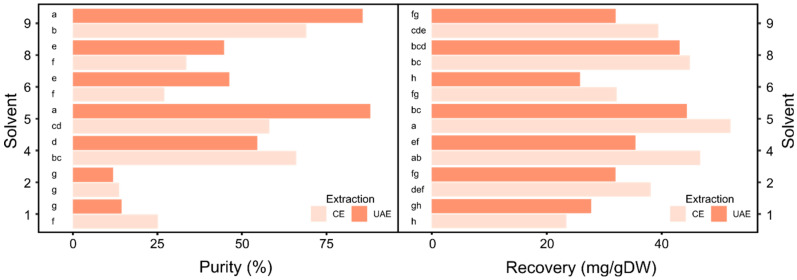
Mean of MPTOX purity (**right**) and recovery (**left**) obtained with UAE and CE from *L. dolomiticum* ARc. Samples with different letters are significantly different at *p* ≤ 0.05 according to Duncan test. Coefficient of variation (CV), purity = 13.3%; recovery = 7.5%. n = 3.

The *L. dolomiticum* adventitious root line used for this experiment produced good amounts of MPTOX-Glc and MPTOX (around 100 and 17 mg/g DW, respectively), using CE with 80% methanol (Figure S2). It has also been reported that the MPTOX-Glc can be easily converted to MPTOX by chemical or enzymatic hydrolysis [14]. From the list of solvents shown in Table 1, the only two solvents capable of extracting a detectable amount of MPTOX-Glc were 1 and 6, possibly due to the high polarity of this molecule. Considering its aglycone MPTOX, solvents 1, 2, 4, 5, 6, 8 and 9 allowed its extraction and quantification, as showed in Figure 3; conversely, solvents 3, 7, 10, 11 and 12 did not.

**Figure 4:**
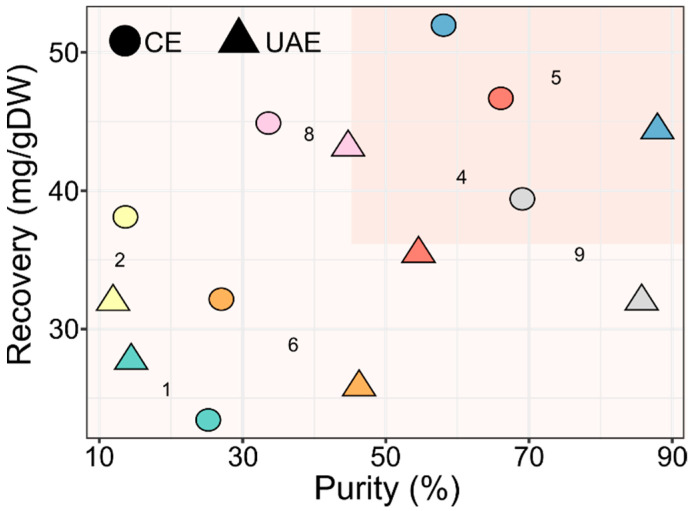
Purity–recovery graph for MPTOX extraction. Purity is expressed as percentage (%), recovery as milligrams for gram of dry weight (mg/g DW). In the top-right section, extractions above the average with the solvent employed are shown.

The two-way ANOVA on the MPTOX data revealed that both factors (solvent and extraction method) and their interaction had a significant effect (Table S2). As regards the purity the two extraction methods, different results were obtained depending on the solvent used. In fact with solvent 2, no difference was found, whereas when using solvents 5, 6, 8 and 9, it was preferable to use UAE, and for solvents 1 and 4, CE gave better performances (see Figure 3 for Duncan test). On the contrary, for MPTOX recovery, apart from solvent 1, the method that allowed obtaining higher values was CE (see Figure 3 for Duncan test). The average values of purity and recovery for MPTOX were 45.8% and 36.5 mg/g DW, respectively (Figure 4).

The solvents that gave purity and recovery higher than average were solvent 5 with both extraction methods and solvents 4 and 9 with CE. In particular, solvent 5 allowed a balance between purity and recovery, reaching values of 87.9% and 44.4 mg/g DW with UAE and of 58.1% and 51.9 mg/g DW with CE. The recovery achieved by CE with this solvent was about two times that obtained using methanol. Moreover, the recovered molecule by UAE was 84% purer when extracted with this solvent compared to the molecule extracted with methanol. Regarding the choice of the extraction method when using solvent 5, UAE maximized the purity, but as shown by the Tuckey test (Figure S6), the MPTOX recovery values after extraction with CE and UAE were not significantly different. Therefore, also in this case UAE is to be preferred, since it allows shortening the extraction time. A similar procedure was used for deoxypodophyllotoxin, a lignan belonging to the ariltetralyn family, obtained from *Anthriscus sylvestris* roots by means of methanol/water and UAE [21]. Finally, the two solvents selected for JB and MPTOX extraction can be easily separated from water, and the molecules of interest can be recovered by solvents evaporation. However, it might be emphasized that these two solvents are partially soluble in water—24% solubility for methyl ethyl ketone and 14% solubility for dimethyl carbonate—which probably helps the solvent to enter the cells. Then, the dissolved lignans can partition and concentrate in the organic phase. Conversely, chloroform has a very low solubility in water (1%) and will hardly enter the cells, which leads to a low recovery.

### 2.4. Optimization of the Operational Conditions of JB Ultrasound-Assisted Extraction (UAE)

To ensure the optimal extraction conditions of JB from *L. austriacum* HR, two parameters were carefully examined, the solvent/water and the liquid/solid ratios. For this screening, after selecting ethyl methyl ketone as the best solvent capable of recovering a high amount of JB with high purity, different combinations of solvent and water, from a 80/20 ratio to a 40/60 ratio, *v*/*v*, and various liquid/biomass ratios (liquid-to-solid ratio, LSR), from 100 up to 400, were studied, thus generating 15 experimental conditions that were investigated. The collected data on recovery and purity were used to generate the response surface for UAE extraction based on two variables, i.e., solvent/water ratio and LSR [21,37,38].

Considering the solvent/water partition of JB measured at 60% of methyl ethyl ketone and LSR 200, the analysis showed only a marginal amount of JB in the water phase, corresponding to just 3.5% of the total recovery, with a purity as low as 20%, while the majority of JB was found in the organic fraction (96.5%) and was easily recoverable by evaporation of methyl ethyl ketone. Therefore, for this study all the aqueous fractions were not measured.

The experimental results were used to generate a response surface for recovery and purity using a generalized additive model (GAM) to fit the data. This methodology is an extension of the generalized linear model which incorporates linear and nonlinear forms of the predictors and estimates these smooth relationships at once, making them more flexible while retaining much of their interpretability [39]. The same innovative approach was used to interpret anthocyanin production in grapevine cell cultures [40]. Since it is required to set the kind of smoothing splines, it is necessary to study the optimal number of knots. Three models were built for the purity and the recovery, with different values of the knots (Tables S3–S5). For both purity and recovery, the best fit was obtained using a combination of knots, k = 3,5 (see Table S4 for all the predicted values). For the purity of JB, the model showed an R^2^adj of 0.885 and an average relative deviation between the experimental and the predicted values of only 0.47%.

**Figure 5:**
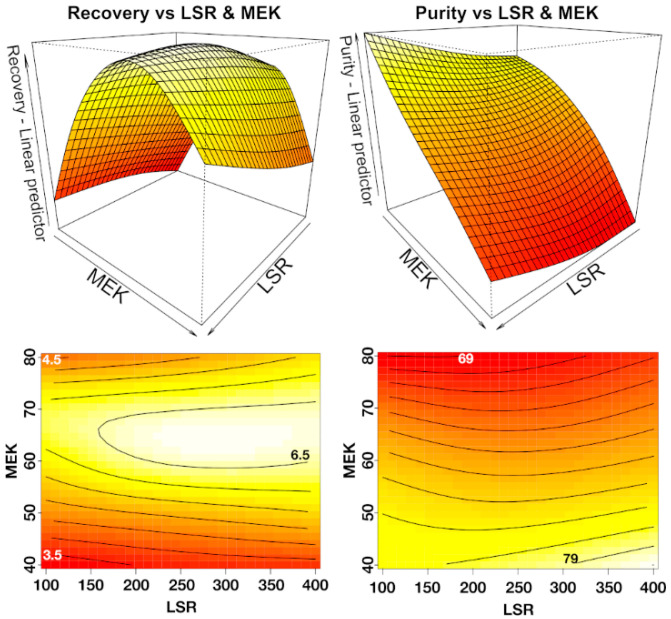
Response surface (**top**) and contour plot (**bottom**) of the recovery (**left**, mg/g DW) and purity (**right**, %) of JB with the combined effect of LSR and methyl ethyl ketone.

In contrast, for the recovery of JB, the R^2^adj was 0.805 with an average relative deviation of 2.77%, demonstrating that the models were reliable and accurate, but recovery was slightly less predictive than purity. The response surface and contour plot for the purity and recovery of JB are shown in Figure 5. From the fitted model, the maximum recovery (6.79 mg/g DW) was obtained using 64.83% methyl ethyl ketone and an LSR of 286. The maximum purity (80.01%) was obtained using 40% methyl ethyl ketone and LSR 400. Looking at the contour plot for recovery and purity, it can be noted that the contour lines are mainly horizontal, along the LSR axis. Since the gradient is normal (perpendicular) to the contour line, and along them the change in recovery or purity is zero, it can be easily assumed that the variation of LSR is less significant than that of the percentage of methyl ethyl ketone for the recovery and purity of JB.

A detailed knowledge of the relationship among purity, recovery, LSR and percentage of organic solvent for the extraction of JB, would allow building a graph that directly links purity and recovery.

**Figure 6:**
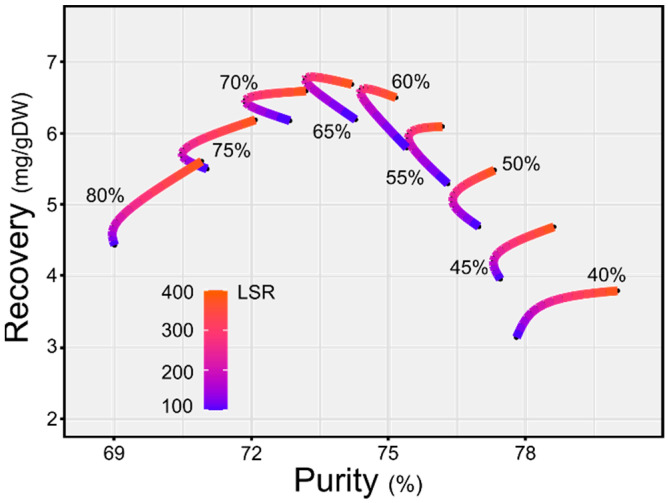
Predicted purity–recovery graph for JB extraction from *L. austriacum* HR with a ethyl methyl ketone/water mixture. Values were obtained with two models for LSR ranging from 100 to 400 and different solvent/water ratios, from 40 to 80%.

Figure 6 depicts the direct empirical relation between recovery and purity obtained for the extraction process of JB from *L.* *austriacum* HRc. The bowed shape of the image suggests that not all combinations of recovery and purity are possible, and three different areas can be identified: low purity with low recovery, medium purity with high recovery and high purity with low recovery. The low purity with low recovery area in Figure 6 is the least interesting and corresponds to high concentrations of methyl ethyl ketone. This indication is noteworthy since it can affect the cost of the process. Decreasing the solvent/water ratio to 65/35 led to the maximum recovery, with a purity around 73%. In particular, it can be noted that this curve is extremely bent, suggesting a minimal influence of LSR. From an industrial point of view, this observation is important because it affects the economics of the process. Moving to the right side of the graph, at higher purity, the solvent/water ratio decreases together with the recovery, and the individual curve becomes more stretched out, presenting marked differences as a function of LSR. The solvent, the solvent/water ratio and the LSR have to be chosen not only on a performance basis but also on the basis of a techno-economic assessment of the process.

## 3. Material and Methods

### 3.1. Plant Material

Seeds of *L. dolomiticum* were purchased from Jelitto (Jelitto Staudensamen, Germany), *L. austriacum* was obtained from USDA (U.S. Department of Agriculture). The seeds were surface-sterilized in 70% ethanol for 1 min and then in a solution of sodium hypochlorite 1% (*v*/*v*) for 5 min, washed 5 times with sterilized water and then germinated in Murashige and Skoog Basal Salt medium 22 at 22 °C in dark conditions. After one week, the seedlings were placed at 25 °C for one month, under 16 h of light and 8 h of darkness. The induction of hairy root cultures from *L. austriacum* and adventitious root cultures from *L. dolomiticum* is described in the Supplementary Data.

### 3.2. Lignans Extraction

Lignans extraction was performed from powdered lyophilized tissues. We mixed 0.05 g of lyophilized roots with 20 mL of different solvents (Table 1, LSR, liquid/solid ratio = 400). Two different types of extraction were evaluated: conventional extraction (CE) and ultrasound-assisted extraction (UAE). CE was performed by means of maceration for 16 h in a rotary shaker at room temperature. UAE was performed using the Omni Sonic Ruptor Ultrasonic Homogenizer 250 (Omni International) for 5 min at 50% power. After extraction, the powered roots were separated by filtration; the organic phase containing lignans, if present, was separated from the aqueous phase, diluted and analyzed by HPLC.

### 3.3. JB and MPTOX HPLC Analysis

Chromatographic analyses were conducted using a Jasco instruments equipped with photodiode array detector. Separation was performed using a Synergi polar RP 80 Å (250 mm × 4.60 mm. 4 μm. Phenomenex). The recovery of JB and MPTOX is expressed as mg/g DW; the purity of JB and MPTOX, expressed as a percentage of the total area, was also determined from the HPLC chromatograms, with integration from 10 to 30 min to avoid the solvent signals. All the details of the HPLC analysis are reported in the Supplementary data.

### 3.4. Optimization of JB Purity and Recovery

Once the best solvent and the best extraction method for JB were established, different liquid/solid ratios and different percentages of solvent were studied to maximize purity and recovery. The first variable (LSR, mL/gDW) was studied choosing three levels (100, 200 and 400); the second one (% solvent) was studied using five levels (40, 50, 60, 70 and 80) for a total of 15 experiments, each one repeated three times (n = 45). The response surface was obtained using the mgcv library for R software [41], which utilizes a generalized additive model (GAM).

### 3.5. Statistical Analysis

The results were subjected to statistical analysis by means of R (as shown in the Supplementary Data).

## 4. Conclusions

For the first time, several green solvents were used in a mixture with water for the extraction of two lignans belonging to the arylnaphthalene and aryltetralin types from in vitro tissue cultures. In terms of purity and recovery, the results showed that ethyl methyl ketone and dimethyl carbonate were the optimal media for extracting justicidin B and 6-methoxypodophyllotoxin, respectively, and when compared to conventional maceration, ultrasound-assisted extraction had some advantages. Using the response surface method, the ideal experimental conditions for extracting justicidin B from *L. austriacum* hairy roots with methyl ethyl ketone were found. The models developed are reliable and accurate for estimating justicidin B purity and recovery. A new type of graph that comprises purity, recovery, LSR and solvent composition is proposed to simplify the choice of the optimal operational conditions.

## Figures and Tables

**Table 1:** List of solvents and solvent/water ratios employed.

#	Solvents	Ratio (*v*/*v*)
1	Methanol	80/20
2	1,3-dioxolane	80/20
3	1,3-dioxolane	100/0
4	Ethyl methyl ketone	80/20
5	Dimethyl carbonate	80/20
6	3-methoxy-3-methylbutan-1-ol	80/20
7	3-methoxy-3-methylbutan-1-ol	100/0
8	Propylene carbonate	80/20
9	Chloroform	80/20
10	1-butanol	80/20
11	Menthol: decanoic acid (1:2, molar ratio)	80/20
12	Menthol: dodecanoic acid (2:1, molar ratio)	80/20

## Data Availability

Not applicable.

## Supplementary Materials

The following supporting information can be downloaded from https://www.mdpi.com/article/10.3390/molecules27092732/s1, *L. austriacum* hairy root cultures (HRc) induction; *L. dolomiticum* adventitious root culture (ARc) induction; JB and MPTOX HPLC quantification; Figure S1: *L. austriacum* hairy root cultures and HPLC chromatogram of lignans extract; Figure S2: *L*. *dolomiticum* adventitious root cultures and HPLC chromatogram of lignans extract; Statistical report: justicidin B from HRc cultures; Table S1: ANOVA on purity and recovery of JB data; Figure S3: Tukey HSD post hoc test for JB purity data; Figure S4: Tukey HSD post hoc test for JB recovery data, 6-methoxypodophyllotoxin (MPTOX) from Arc; Table S2: Two-way ANOVA with interaction on MPTOX purity and recovery data; Figure S5: Tukey HSD post hoc test for purity of MPTOX data; Figure S6: Tukey HSD post hoc test for recovery of MPTOX data; Optimization of JB purity and recovery; Table S3: Results of generalized additive model (GAM) for JB purity and recovery; Diagnosis of GAM models; Table S4: Diagnostic parameters for purity model; Figure S7: Diagnostic plots for purity model; Table S5: Diagnostic parameters for recovery model; Figure S8: Diagnostic plots for recovery model; Table S6: Experimental data and predicted values for JB purity (%) and recovery;. References: [42,43,44,45,46,47].
