# Supplementary material for: Optimized Ultrasound-Assisted Extraction of Lignans from Linum Species with Green Solvents
Source: Molecules. 2022 Apr 23;27(9):2732. doi: 10.3390/molecules27092732 (PMC9104071; doi:10.3390/molecules27092732)
Supplement: Supplementary file 1 [file molecules-27-02732-s001.zip › molecules-1692027-supplementary.pdf]

Electronic supporting information

Optimized ultrasound-assisted extraction of lignans from *Linum* species with green solvents

Michela Alfieri[a], Iride Mascheretti[b], Roméo A. Dougué Kentsop[b], Monica Mattana[b], Marina Laura[c] and Gianluca Ottolina[a]*

[a] *Institute of Chemical Sciences and Technologies "Giulio Natta", National Research Council, Via Mario Bianco 9, 20131 Milan, Italy.*
[b] *Institute of Agricultural Biology and Biotechnology, National Research Council, Via Bassini 15, 20133 Milan, Italy.*
[c] *CREA Research Centre for Vegetable and Ornamental Crops (CREA OF), Corso degli Inglesi 508, 18038 Sanremo, Italy.*

Summary list

Experimental 1
L. austriacum hairy root cultures (HRc) induction 1
L. dolomiticum adventitious root culture (ARc) induction 1
JB and MPTOX HPLC quantification 1
Figure S1 2
Figure S2 2

Statistical report 3
Justicidin B from HRc cultures 3
Table S1 3
Figure S3 4
Figure S4 5
6-methoxypodophyllotoxin (MPTOX) from ARc 6
Table S2 6
Figure S5 7
Figure S6 8
Optimization of JB purity and recovery 9
Table S3 9

Diagnosis of GAM models 10
Table S4 10
Figure S7 10
Table S5 11
Figure S8 11
Table S4 12

References 12

***L. austriacum* hairy root cultures (HRc) induction**

To induce hairy roots formation, leaf and stem explants from in vitro seedlings of *L. austriacum* were incubated with *Agrobacterium rhizogenes* strain ATCC 15,834 (Kajala et al., 2014). The *A. rhizogenes* was grown overnight at 28 °C in yeast mannitol broth (YMB: 0.5 g/L K_2HPO_4, 0.2 g/L $MgSO_4 \cdot 7H_2O$, 0.1 g/L NaCl, 10 g/L mannitol and 0.4 g/L yeast extract) supplemented with 200 µM acetosyringone (AS). The cultures were then pelleted by centrifugation and resuspended in MS medium supplemented with 200 µM AS and adjusting OD600 to 0.6. The explants were incubated in the *Agrobacterium* solution for 20 min at room temperature and then blotted on sterile filter paper. The explants were co-cultivated with *Agrobacterium* on solid co-cultivation media (MS medium with 3% (w/v) sucrose and 0.8% (w/v) agar, pH 5.7) for 3 days at 24 °C in the dark and then were transferred on the same media supplemented with 100 µg/mL cefotaxime. After about two weeks, calli were formed from all explants and then from two weeks-old calli several hairy roots emerged. Individual putative hairy roots of approximately 3 cm long were excised and immediately transferred on MS medium supplemented with 50 µg/mL cefotaxime. All the hairy roots were maintained separately as independent lines. The roots were subcultured every 15 days in the presence of 50 µg/mL cefotaxime until the complete elimination of bacteria. To initiate hairy root suspension culture (HRc), 0.8 g FW of hairy roots were transferred to 250 mL Erlenmeyer flasks containing 50 mL of MS medium. HRc were grown at 25 °C on a gyratory shaker at 110 rpm under permanent dark conditions. All the media and components for in vitro cultures were purchased by Duchefa-Biochemie, Haarlem, The Netherlands. Genomic DNA from 10 independent lines of hairy roots were extracted using DNeasy Plant Mini Kit (Qiagen, Hilden, Germany) according to the manufacturer's instructions. The genomic DNA extracted was used as template to amplify a fragment of rolC gene of *A. rhizogenes*. The primer sequences are as follows: Forward: 50-CGACCTGTGTTCTCTCTTTTTCAAGC-30 and Reverse: 50-GCACTCGCCATGCCTCACCAACTCACC-30. The lines that were positive in PCR analysis of the rolC gene were checked for the absence of A. rhizogenes DNA by a PCR targeting the 326 bp fragment of virC1 (bacterial chromosome), following (Vaira et al., 1995).

***L. dolomiticum* adventitious root culture (ARc) induction**

ARc were generated from leaves collected from one month-old plantlets *L. dolomiticum*. The sterile leaf explants were laid horizontally on Petri dishes with solid (0.8% agar) MS supplemented with vitamins, 2 mg/L α-naphthalene acetic acid (NAA), 0.4 mg/L kinetin and 30 mg/L of sucrose. The pH was adjusted to 5.8 using KOH solution. After 4 weeks, the length of the root segments was between 0.8-1.3 cm. In order to increase the biomass production, roots were individually transferred to half strength MS medium supplemented with 20 mg/L of sucrose 0.5 mg/L indole-3-butyric acid (IBA) and 0.1 mg/L indole-3-acetic acid (IAA) (shortened from now on as MS-II) and the roots were subcultured every month.

JB and MPTOX HPLC quantification

To quantify JB from *L. austriacum* extracts the mobile phase consisted of water containing 0.1 % formic acid (v/v) (A) and acetonitrile containing 0.1 % formic acid (v/v) (B). Gradient conditions were as follows: 0-70 % B for the first 26 min, from 70 to 0 % B in 1 min and 0 % B up to 30 min. The flow rate was 1.0 mL/min. The wavelength used for the integration of the signals was 254 nm. JB retention time (Rt) 22.6 min. The JB quantification was performed with standard calibration curve obtained using seven standard dilutions ranging from 9.13 to 86.00 µg/mL. Each standard solution was injected in duplicate. The linear regression equation was carried out by plotting the peak areas against the injected amounts of standard compounds. giving a R^2 of 0.9990. The limits of detection (LOD) and the limits of quantification (LOQ) were determined as: LOD = 1.94 µg/mL and LOQ = 6.45 µg/mL.

To quantify MPTOX from *L. dolomiticum* extracts the mobile phase consisted of water (A) and acetonitrile (B). Gradient conditions were as follows: 0 -20% B for the first 15 min, 20 -70% B from 15 to 25min, 70% B for 3min and 70 -0% B up to 30 min. The flow rate was 1.0 mL/min. The wavelength used for the integration of the signals was 250 nm. MPTOX retention time (Rt) 24.2 min. The MPTOX quantification was performed with standard calibration curve obtained using five standard dilutions ranging from 107 µg/mL to 533 µg/mL. Each standard solution was injected in duplicate. The linear regression equation was carried out by plotting the peak areas against the injected amounts of standard compounds. giving a R^2 of 0.9985. The limits of detection and the limits of quantification were determined as: LOD= 23.1 µg/mL; LOQ = 76.9 µg/mL.

Figure S1 *L. austriacum* hairy root cultures and HPLC chromatogram of lignans extract in 80% methanol, with the peak at Rt = 22.6 min corresponding to JB. For the purity determination, the chromatogram was integrated from 10 min to 30 min.

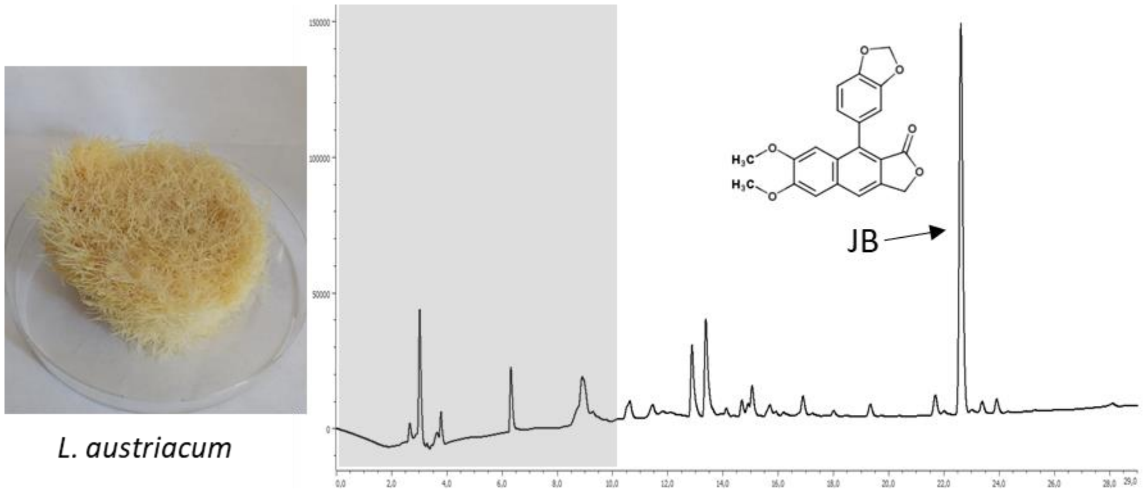

Figure S2 *L. dolomiticum* adventitious root cultures and HPLC chromatogram of lignans extract in 80% methanol, Rt = 19.9 min MPTOX-Glc and Rt = 24.2 min MPTOX. For the purity determination, the chromatogram was integrated from 10 min to 30 min.

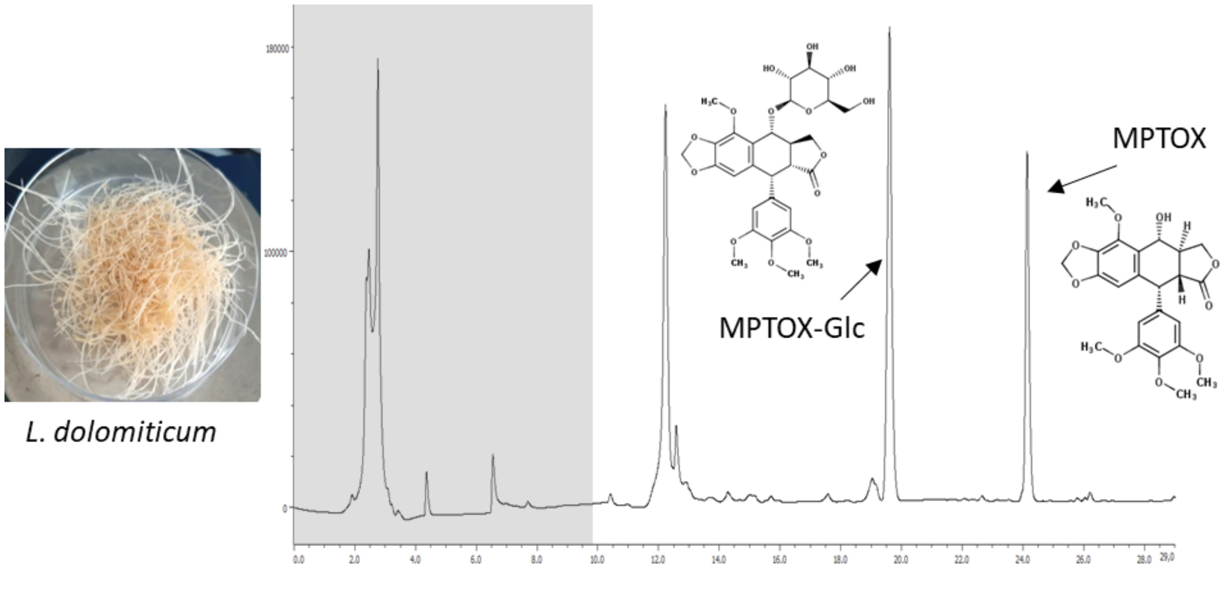

Statistical report

Statistical analysis were performed using R (R Core Team, 2022), library rstatix (Alboukadel Kassambara, 2021), tidyverse (Wickham et al., 2019), agricolae (Felipe de Mendiburu, 2020).

Justicidin B from HRc cultures

Residual analysis was performed to test for the assumptions of the two-way ANOVA. Outliers were assessed by box plot method; normality was assessed using Shapiro-Wilk's normality test and homogeneity of variances was assessed by Levene's test. There were no extreme outliers, residuals were normally distributed ($p > 0.05$) and there was homogeneity of variances ($p > 0.05$).

A one-way or two-way ANOVA was conducted to examine the effects of solvents or extraction methods on JB purity and recovery, as showed in Table S1. Tukey HSD post hoc tests were carried out. All pairwise comparisons were analyzed between the different solvents groups organized by extraction methods; the results are showed in Figure S3 for JB purity data and Figure S4 for JB recovery data. Duncan test was performed considering $p < 0.05$.

Table S1 ANOVA on purity and recovery of JB data

**Purity – Two-way ANOVA with interaction**					
Effect	DFn	DFd	F	p	η^2_g
Solvent	11	48	194.607	7.01e-36	0.978
Extraction	1	48	24.873	8.42e-06	0.341
Solvent x extraction	11	48	6.280	2.71e-06	0.590
**Recovery – One way ANOVA**					
Effect	DFn	DFd	F	p	η^2_g
Solvent	11	60	86.853	1.34 e-32	0.941

DFn, degrees of freedom in the numerator; DFd, degrees of freedom in the denominator; F, F-value; p, p-value; η^2_g, generalized eta squared.

Figure S3 Tukey HSD post hoc test for JB purity data. Only the not significant pairwise comparisons were shown.

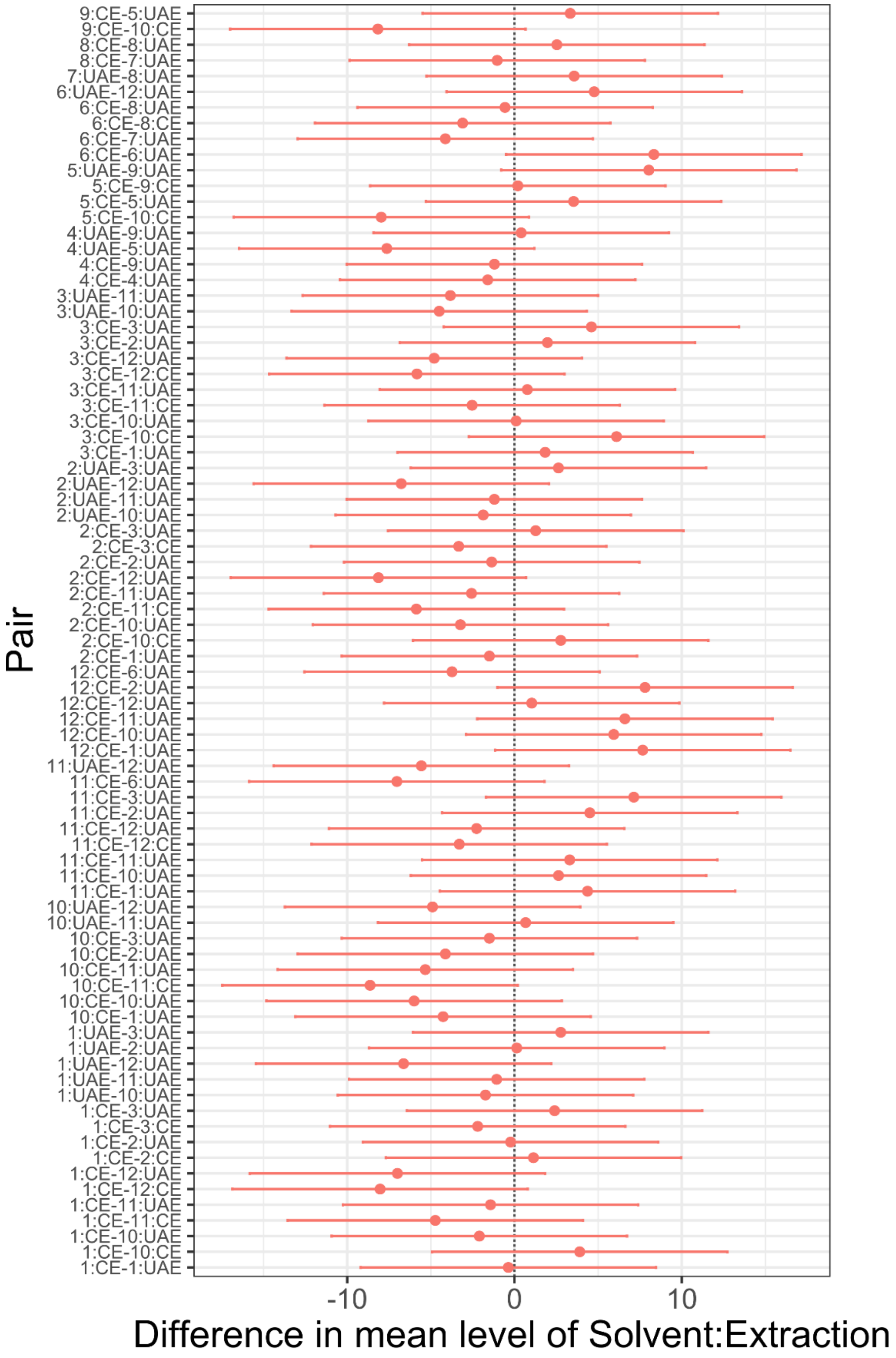

Figure S4 Tukey HSD post hoc test for JB recovery data. Only the not significant pairwise comparisons were shown.

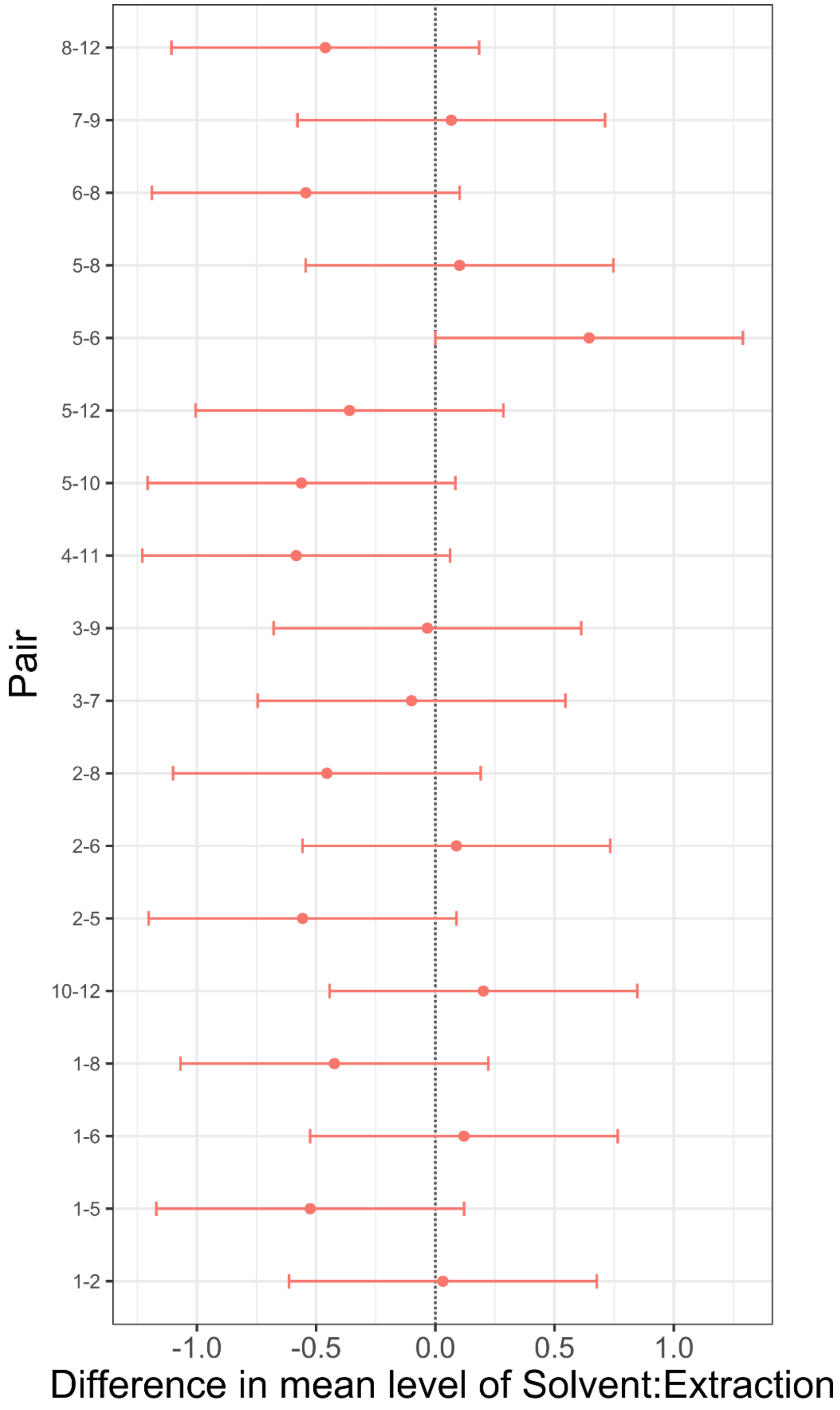

6-methoxypodophyllotoxin (MPTOX) from ARc

Residual analysis was performed to test for the assumptions of the two-way ANOVA. Outliers were assessed by box plot method, normality was assessed using Shapiro-Wilk's normality test and homogeneity of variances was assessed by Levene's test. There were no extreme outliers, residuals were normally distributed ($p > 0.05$) and there was homogeneity of variances ($p > 0.05$).

A one-way or two-way ANOVA was conducted to examine the effects of solvents or extraction methods on MPTOX purity and recovery, as showed in Table S2. Tukey HSD post hoc tests were carried out. All pairwise comparisons were analyzed between the different solvents groups organized by extraction methods; the results are showed in Figure S5 for MPTOX purity data and Figure S6 for MPTOX recovery data. Duncan test was performed considering $p < 0.05$.

Table S2 Two-way ANOVA with interaction on MPTOX purity and recovery data

Purity					
Effect	DFn	DFd	F	p	η_g^2
Solvent	6	28	156.744	3.24e-20	0.971
Extraction	1	28	24.459	3.22e-05	0.466
Solvent x extraction	6	28	15.449	9.64e-08	0.768
**Recovery**					
Effect	DFn	DFd	F	p	η_g^2
Solvent	6	28	33.593	1.52e-11	0.878
Extraction	1	28	24.098	3.56e-05	0.463
Solvent x extraction	6	28	3.247	1.50e-02	0.410

DFn, degrees of freedom in the numerator; DFd, degrees of freedom in the denominator; F, F-value; p, p-value;
η_g^2, generalized eta squared

Figure S5 Tukey HSD post hoc test for purity of MPTOX data. Only the not significant pairwise comparisons were shown.

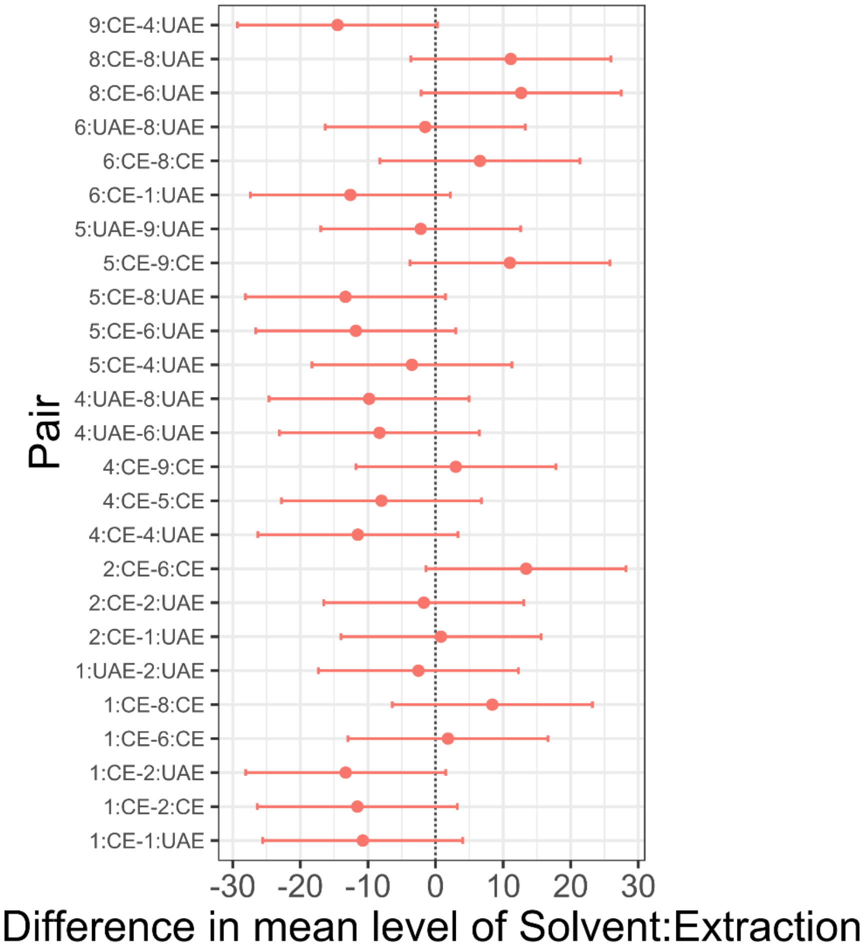

Figure S6 Tukey HSD post hoc test for recovery of MPTOX data. Only the not significant pairwise comparisons were shown.

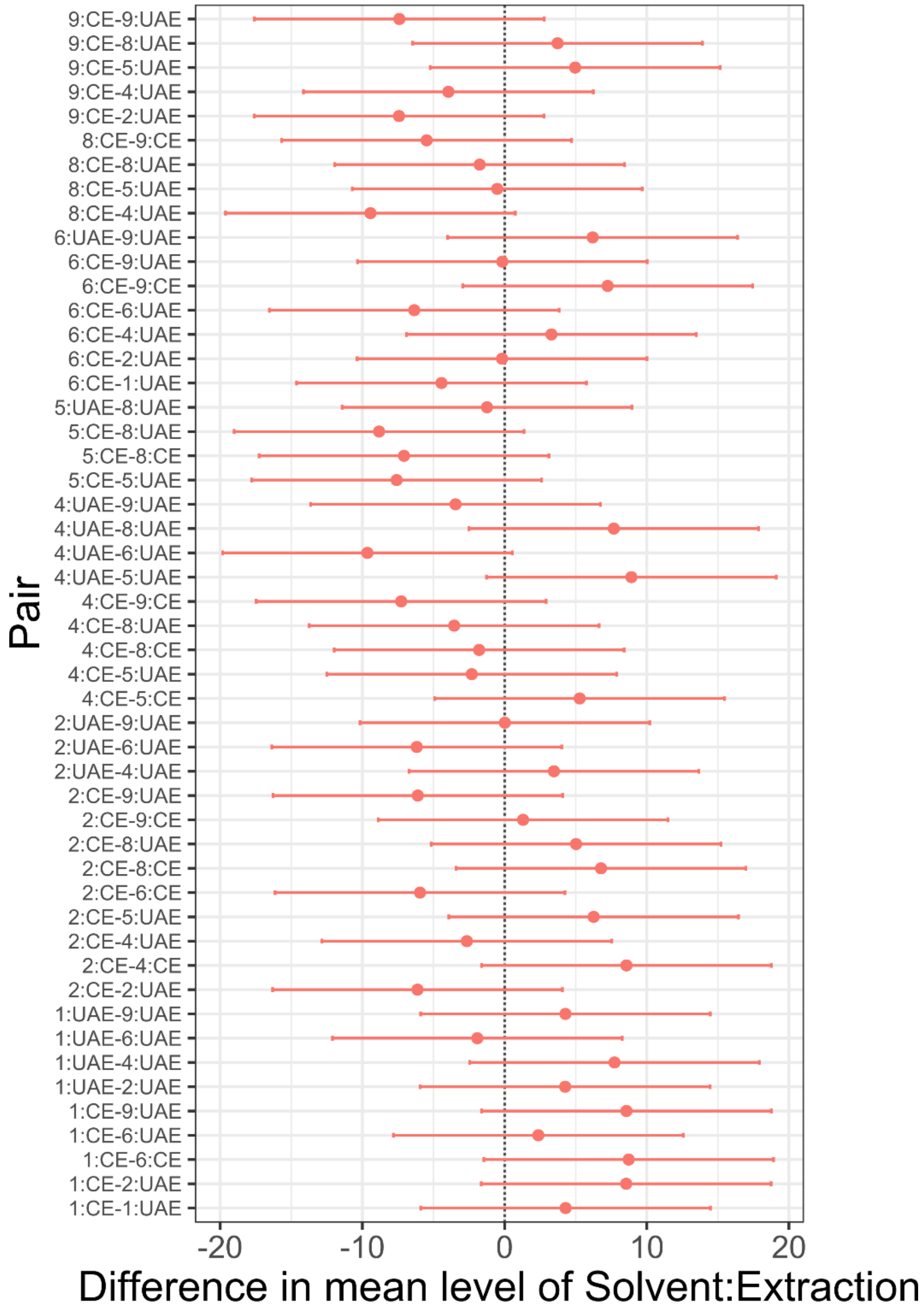

Optimization of JB purity and recovery

The response surface was obtained using the mgcv library for R software (Wood, 2021), which utilize generalize additive model (GAM).

Table S3 Results of generalized additive model (GAM) for JB purity and recovery, n = 45

	R^2 adj	Deviance explained	GCV	Scale est.	AIC
**Purity**					
k = 3, 3	0.883	90.0%	1.7091	1.4313	152.4871
k = 3, 4	0.885	90.3%	1.7298	1.4169	152.6859
k = 3, 5	0.885	90.5%	1.7417	1.4111	152.8128
**Recovery**					
k = 3, 3	0.758	79.0%	0.46208	0.39257	93.83046
k = 3, 4	0.795	82.9%	0.41016	0.33292	87.77053
k = 3, 5	0.805	84.6%	0.41004	0.31647	86.81181

k, knots; R^2 adj, R^2 adjusted; GCV, generalized cross validation; AIC, Akaike information deviation

Diagnosis of GAM models

-Purity, k=3,5
Model formula: Purity ~ te(LSR, MEK, k = c(3, 5))

Table S4 Diagnostic parameters for purity model.

Parametric coefficient				
	Estimate	*Std. Error*	*t value*	*Pr(>|t|)*
Intercept	74.5756	0.1771	421.1	<2e-16
**Approximate significance of smooth terms**				
	edf	*Ref.df*	*F*	*p-value*
te(LSR,MEK)	7.542	9.44	36.08	<2e-16

te, tensor; LSR, liquid-solid ratio; MEK, ethyl methyl ketone; edf, equivalent degrees of freedom; Ref.df, reference number of degrees of freedom.

Figure S7 Diagnostic plots for purity model.

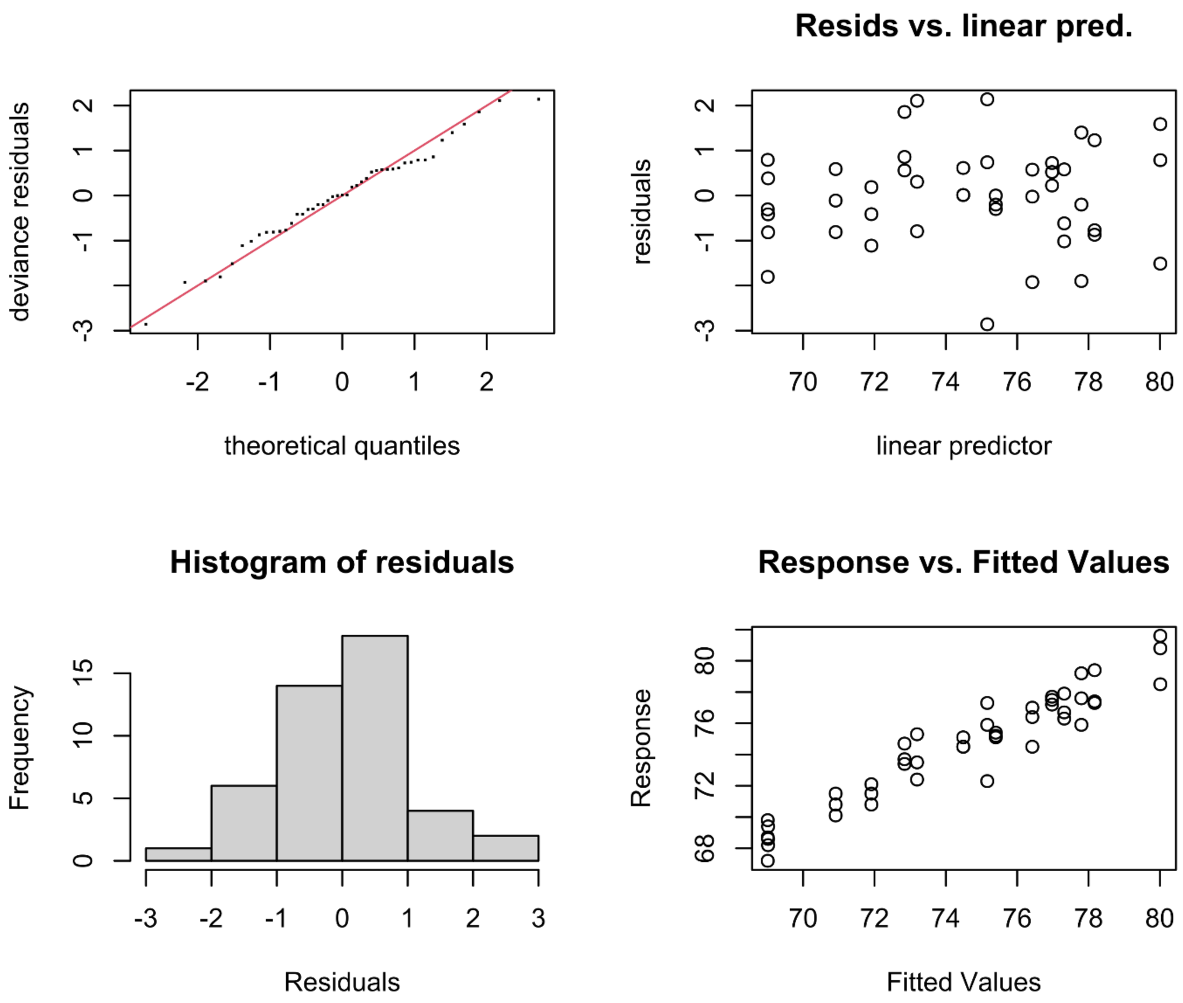

Recovery, k= 3,5

Model formula: Recovery ~ te(LSR, MEK, k = c(3, 5))

Table S5 Diagnostic parameters for recovery model

Parametric coefficient				
	Estimate	*Std. Error*	*t value*	*Pr(>|t|)*
Intercept	5.21956	0.08386	62.24	<2e-16
**Approximate significance of smooth terms**				
	edf	*Ref.df*	*F*	*p-value*
te(LSR,MEK)	9.269	11.39	15.87	<2e-16

te, tensor; LSR, liquid-solid ratio; MEK, ethyl methyl ketone; edf, equivalent degrees of freedom; Ref.df, reference number of degrees of freedom.

Figure S8 Diagnostic plots for recovery model.

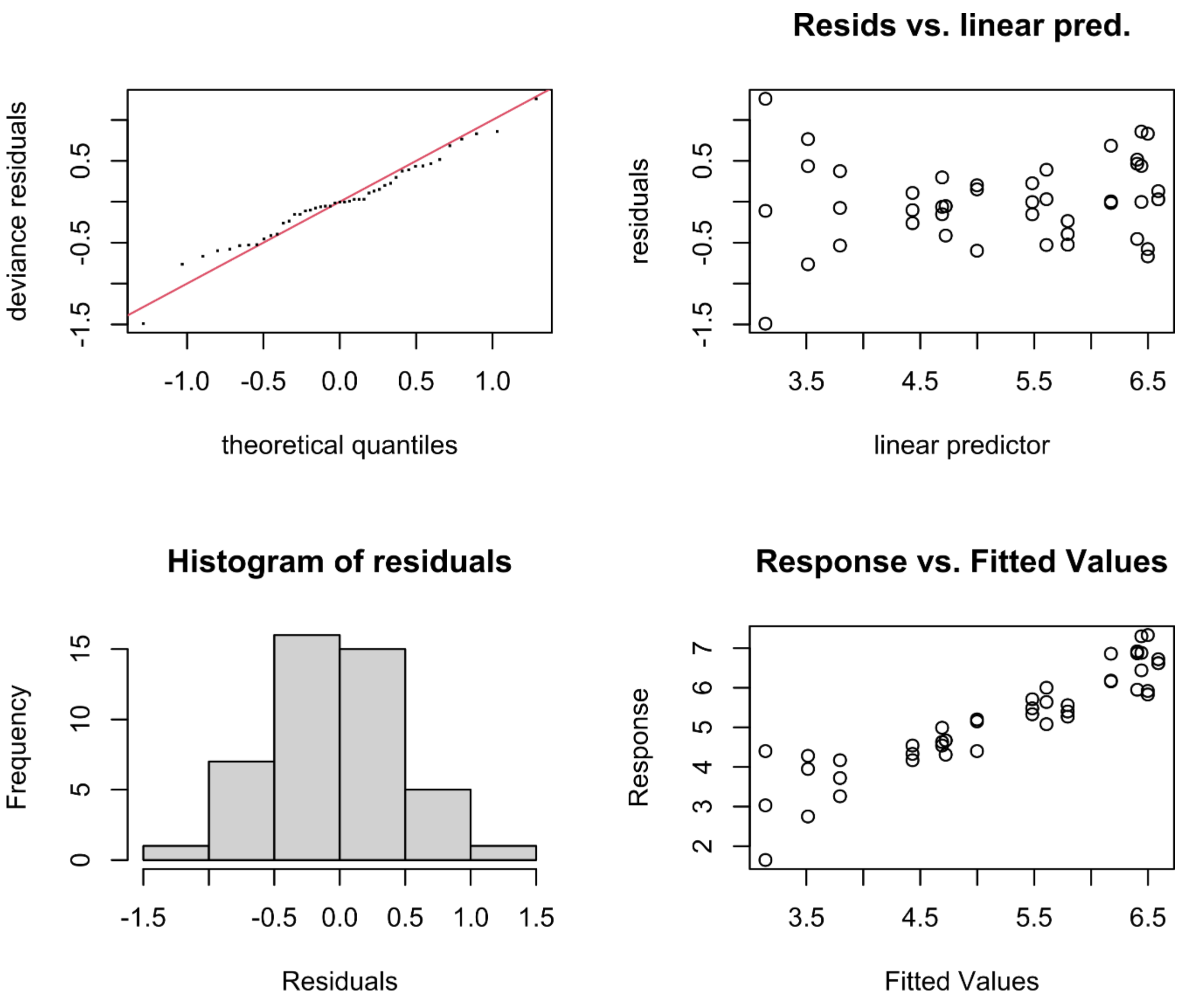

Table S4 Experimental data and predicted values for JB purity (%) and recovery (mg/gDW)

		Experimental				Predicted			
		Purity		Recovery		Purity		Recovery	
LSR	MEK	Mean	SD	Mean	SD	Fit	SE.Fit	Fit	SE.Fit
100	40	77.57	1.65	3.03	1.38	77.80	0.60	3.14	0.29
200	40	78.03	1.19	3.66	0.81	78.17	0.56	3.51	0.25
400	40	80.30	1.61	3.72	0.46	80.01	0.62	3.80	0.31
100	50	77.47	0.25	4.72	0.24	76.98	0.46	4.69	0.26
200	50	75.97	1.31	4.92	0.48	76.42	0.43	5.00	0.23
400	50	76.97	0.83	5.51	0.19	77.32	0.47	5.48	0.28
100	60	75.23	0.15	5.41	0.15	75.40	0.46	5.79	0.26
200	60	74.70	0.35	6.87	0.43	74.49	0.44	6.44	0.23
400	60	75.17	2.58	6.36	0.84	75.16	0.48	6.50	0.27
100	70	73.93	0.68	6.40	0.40	72.84	0.46	6.17	0.26
200	70	71.47	0.65	6.58	0.55	71.91	0.43	6.41	0.23
400	70	73.73	1.46	6.65	0.58	73.19	0.47	6.59	0.28
100	80	68.57	1.31	4.35	0.19	69.01	0.60	4.43	0.29
200	80	68.73	0.61	4.55	0.21	69.02	0.56	4.72	0.25
400	80	70.80	0.70	5.57	0.46	70.91	0.62	5.61	0.31

LSR, liquid solid ratio; MEK, methyl ethyl ketone; SD, standard deviation; SE, standard error

References

Alboukadel Kassambara (2021). rstatix: Pipe-Friendly Framework for Basic Statistical Tests. R package https://CRAN.R-project.org/package=rstatix

Felipe de Mendiburu (2020). agricolae: Statistical Procedures for Agricultural Research. R package https://CRAN.R-project.org/package=agricolae

R Core Team (2022). R: A language and environment for statistical computing. R Foundation for Statistical Computing, Vienna, Austria. https://www.R-project.org/

Wickham et al., (2019). Welcome to the tidyverse. Journal of Open Source Software, 4(43), 1686, https://doi.org/10.21105/joss.01686

Kajala, K.; Coil, D. A.; Brady, S. M. Draft Genome Sequence of Rhizobium Rhizogenes Strain ATCC 15834. Genome Announc. 2014, 2 (5). https://doi.org/10.1128/genomeA.01108-14.

Vaira, A. M.; Semeria, L.; Crespi, S.; Lisa, V.; Allavena, A.; Accotto, G. P. Resistance to tospoviruses in Nicotiana benthamiana transformed with the N Gene of tomato spotted wilt virus: correlation between transgene expression and protection in primary transformants. *Mol. Plant-Microbe Interact. MPMI* **1995**, *8* (1), 66–73. https://doi.org/10.1094/mpmi-8-0066.

Simon Wood, 2021. mgcv: Mixed GAM Computation Vehicle with Automatic Smoothness Estimation. R package https://cran.r-project.org/web/packages/mgcv/index.html
